# Novel Biomarkers Distinguishing Active Tuberculosis from Latent Infection Identified by Gene Expression Profile of Peripheral Blood Mononuclear Cells

**DOI:** 10.1371/journal.pone.0024290

**Published:** 2011-08-31

**Authors:** Chanyi Lu, Jing Wu, Honghai Wang, Sen Wang, Ni Diao, Feifei Wang, Yan Gao, Jiazhen Chen, Lingyun Shao, Xinhua Weng, Ying Zhang, Wenhong Zhang

**Affiliations:** 1 Department of Infectious Diseases, Huashan Hospital, Fudan University, Shanghai, China; 2 State Key Laboratory of Genetic Engineering(SKLGE), Institute of Genetics, School of Life Sciences, Fudan University, Shanghai, China; 3 Department of Molecular Microbiology and Immunology, Bloomberg School of Public Health, Johns Hopkins University, Baltimore, Maryland, United States of America; 4 Institutes of Biomedical Sciences, Fudan University, Shanghai, China; University of Hyderabad, India

## Abstract

**Background:**

Humans infected with *Mycobacterium tuberculosis* (MTB) can delete the pathogen or otherwise become latent infection or active disease. However, the factors influencing the pathogen clearance and disease progression from latent infection are poorly understood. This study attempted to use a genome-wide transcriptome approach to identify immune factors associated with MTB infection and novel biomarkers that can distinguish active disease from latent infection.

**Methodology/Principal Findings:**

Using microarray analysis, we comprehensively determined the transcriptional difference in purified protein derivative (PPD) stimulated peripheral blood mononuclear cells (PBMCs) in 12 individuals divided into three groups: TB patients (TB), latent TB infection individuals (LTBI) and healthy controls (HC) (n = 4 per group). A transcriptional profiling of 506 differentially expressed genes could correctly group study individuals into three clusters. Moreover, 55- and 229-transcript signatures for tuberculosis infection (TB&LTBI) and active disease (TB) were identified, respectively. The validation study by quantitative real-time PCR (qPCR) performed in 83 individuals confirmed the expression patterns of 81% of the microarray identified genes. Decision tree analysis indicated that three genes of CXCL10, ATP10A and TLR6 could differentiate TB from LTBI subjects. Additional validation was performed to assess the diagnostic ability of the three biomarkers within 36 subjects, which yielded a sensitivity of 71% and specificity of 89%.

**Conclusions/Significance:**

The transcription profiles of PBMCs induced by PPD identified distinctive gene expression patterns associated with different infectious status and provided new insights into human immune responses to MTB. Furthermore, this study indicated that a combination of CXCL10, ATP10A and TLR6 could be used as novel biomarkers for the discrimination of TB from LTBI.

## Introduction

Tuberculosis remains a leading infectious disease worldwide. There were 9.4 million new TB cases with 1.6 million deaths in 2009 and about 2 billion people were latently infected with MTB [1], [2]. Most people infected with MTB remain asymptomatic, termed LTBI, presumably due to human protective immune response [3], [4]. In the United States, 20–30% of close contacts have latent infection [5], [6], while an estimated 15% of Chinese population have latent infection identified by T-cell-based gamma interferon (IFN-γ) release assays (IGRAs) [7]. 10% of LTBI individuals will progress to active TB in their lifetime [1]. However, immunological determinants of host-pathogen interactions resulting in disease progression, latency or clearance remain poorly understood. Although human immune factors are known to play a pivotal role in the control of infection [8], [9], [10], more immunological network signatures with respect to disease, MTB infection or clearance need to be defined [11], [12].

Although QuantiFERON-TB Gold In-tube (Cellestis, Victoria, Australia) and T-SPOT.TB (Oxford Immunotech, UK) based on ESAT-6 and CFP-10 are capable of distinguishing individuals vaccinated with BCG from MTB infection [13], [14], these assays could not discriminate active TB disease from LTBI [15]. Apparently, MTB-specific IFN-γ release is not sufficient to represent the comprehensive immunological changes stimulated by MTB. It is generally accepted that both innate and acquired immune responses play an important role in controlling MTB infection [8], [9]. Nevertheless, when conditions such as HIV infection, immunosuppressive agents or stresses compromise normal immune function, latent infection can be reactivated to clinical disease [16], [17], [18].

Many studies have been done to clarify the cytokine and chemokine responses to MTB-specific antigens that are involved in the progression from latent infection to active disease [19], [20], [21]. However, these studies revealed only limited insight into human resistance or susceptibility to MTB infection. More recently, genome-wide transcriptome analyses have been widely used to explore the complex interaction between human and bacteria [22], [23], [24], [25]. However, results varied due to diverse genetic background of the study population and the inherent complexity of the disease process and the immune response. In addition, most of these microarray studies used the peripheral whole blood without stimulation by MTB-specific antigens, which may lead to interference with other conditions than TB and skew the resulting TB-associated transcription profiles and candidate genes used for discriminating TB from LTBI.

To better understand the immunologic characteristics of different status of TB infection and to identify potential immune biomarkers that could discriminate active disease from latent infection, we performed genome-wide transcription analysis of PPD-stimulated PBMCs from subjects with TB, LTBI and HC, and identified unique transcript profiles in individuals with tuberculosis infection and active disease. The identified signatures will not only provide new insight into the immune mechanisms involved in MTB infection but also furnish new biomarkers which may distinguish active TB patients from LTBI individuals.

## Results

### Identification of differences in gene expression profiling of PBMCs in response to PPD in TB, LTBI, and HC groups

Based on our hypothesis that PBMCs from individuals with different MTB infectious status could exhibit distinct transcription profiles when stimulated by PPD, we isolated PBMCs from 12 subjects (TB = 4, LTBI = 4, HC = 4) and then stimulated them with or without PPD for 4 hours. Using Whole Human Genome Oligo Microarray (Agilent), we determined the fold changes of gene expression levels regulated by PPD in the PBMCs. The ratios of the fold changes were compared among three pair-wise comparisons (LTBI vs. HC; TB vs. LTBI; and TB vs. HC). Transcriptional profiles of LTBI and HC groups exhibited relatively similar patterns with 94 differentially expressed genes being observed, while TB groups exhibited much more differentially expressed genes when compared with LTBI and HC groups (n = 239 and 286, respectively; Table 1). Differentially expressed genes in these three pair-wise comparisons with ratio >4 were present in Table 2, which were largely dominated by genes encoding chemokines, cytokines and receptors.

**Table 1:** Gene expression ratios among different groups and their comparisons in PPD-stimulated PBMCs by microarray analysis with *P* value <0.05.

	ratio>8	8>ratio>4	4>ratio>2
**Pair-wise comparisons**			
LTBI/HC	2	3	26
HC/LTBI	0	0	63
TB/LTBI	0	3	136
LTBI/TB	1	4	142
TB/HC	9	7	98
HC/TB	1	0	124
**Different infectious status**			
TB&LTBI/HC	2	7	26
HC/TB&LTBI	0	0	40
TB/LTBI&HC	0	9	101
LTBI&HC/TB	1	1	137

**Table 2:** Significantly regulated genes in PPD-stimulated PBMCs in pair-wise comparisons with ratio >4.

GeneSymbol	Means		Ratio	Regulation	t-test
**LTBI/HC**	**LTBI**	**HC**			***P*** ** value**
IL2	72.38	5.65	12.81	up	0.0117
CXCL9	6.58	1.32	4.97	up	0.0284
IFNG	7.09	1.76	4.03	up	0.0002
BATF2	3.39	0.84	4.01	up	0.0201
**TB/LTBI**	**TB**	**LTBI**			***P*** ** value**
BX090755	0.10	1.11	11.51	down	0.0437
DB513559	0.09	0.45	5.29	down	0.0239
SDS	0.07	0.31	4.66	down	0.0225
A_24_P931568	0.88	3.93	4.49	down	0.0010
WDR69	0.19	0.83	4.25	down	0.0139
MYO1B	4.75	1.12	4.25	up	0.0014
HMGA2	4.97	1.20	4.13	up	0.0054
AF113012	1.74	0.43	4.02	up	0.0202
**TB/HC**	**TB**	**HC**			***P*** ** value**
IL2	128.76	5.65	22.80	up	0.0076
IFNG	24.46	1.76	13.92	up	0.0002
CXCL9	16.42	1.32	12.41	up	0.0095
CXCL11	6.64	0.65	10.21	up	0.0383
CXCL10	7.48	0.77	9.69	up	0.0136
BX090755	0.10	0.86	8.91	down	0.0342
CCL8	6.22	0.70	8.90	up	0.0259
UBD	8.01	0.96	8.31	up	0.0285
RSPO3	1.76	0.24	7.19	up	0.0275
BATF2	4.85	0.84	5.74	up	0.0160
Q9BVX4	0.48	0.09	5.62	up	0.0075
SLAMF8	2.83	0.54	5.25	up	0.0195
IL22	4.75	1.14	4.19	up	0.0458
MUCL1	7.00	1.68	4.17	up	0.0069
PDGFRA	5.55	1.36	4.08	up	0.0205

The gene ontology (GO) analysis of differentially expressed genes revealed that genes associated with extracellular component and movement in response to external stimulus were significantly enriched in the comparison of LTBI and HC. Meanwhile, gene categories of extracellular region part, receptor binding, signal transduction, response to stimulus, regulation of immune system process and cell communication were enriched between TB and HC. However, only one GO term with function of response to external stimulus was identified in the comparison between TB and LTBI groups (Table S1).

**Figure 1:**
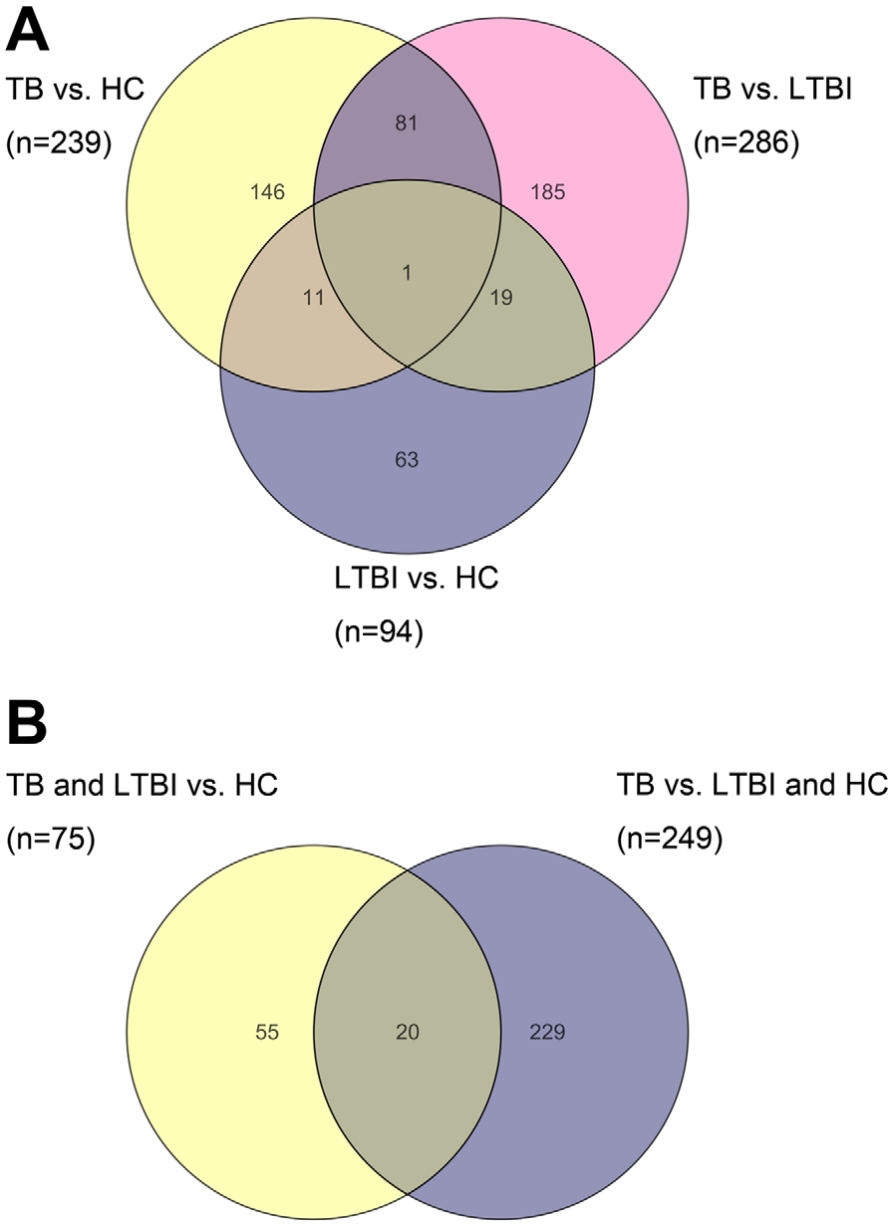
Venn diagram of differentially expressed genes in PBMCs samples following PPD stimulation with *P* value <0.05 by Student's t-test and fold change >2.0 from: A, Pair-wise comparisons between three study groups (LTBI vs. HC; TB vs. HC; TB vs. LTBI); B, Transcription profiles of tuberculosis infection and active disease (TB&LTBI group vs. HC group; TB group vs. LTBI&HC group). The total numbers of genes in panel A and B were 506 and 304 respectively.

Venn diagram of these differentially expressed genes in three pair-wise comparisons showed that most genes were unique in each pair-wise comparison, with 67% (63/94) in LTBI vs. HC, 65% (185/286) in TB vs. LTBI, and 61% (146/239) in TB vs. HC (Figure 1A). After deducting the number of shared genes among the comparisons, a total of 506 differentially expressed genes were identified. Comparisons of TB with the other two groups (TB vs. LTBI and TB vs. HC) shared the biggest number of differentially expressed genes (n = 82), which were mainly involved in pathways of signaling molecules and interaction, cancers, signal transduction, and cell communication. Among the 506 genes, IFN-γ was shared in all three pair-wise comparisons (Figure 1A).

**Figure 2:**
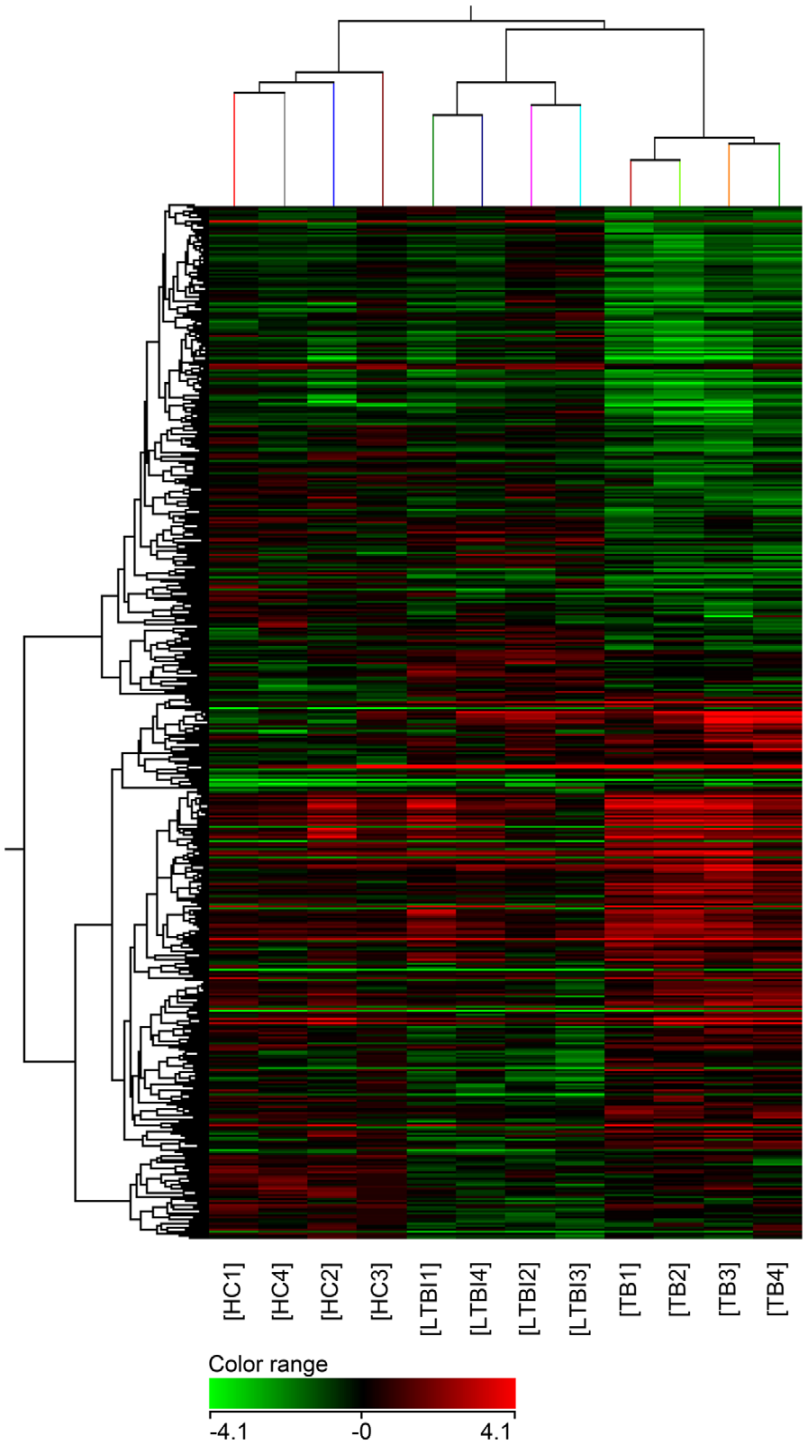
Unsupervised hierarchical cluster analysis of 506 differentially expressed genes in the pair-wise comparisons. There are 4 samples in each group. Pseudocolors indicate differential expression (red, up-regulation; green, down-regulation; black, no change in expression).

In order to determine whether the expression patterns of this 506-transcript profile above could indeed reflect the different status of MTB infection, unsupervised hierarchical cluster analysis was performed. It showed that 12 individuals were successfully clustered into three groups, and each group matched exactly to the corresponding grouping of TB, LTBI and HC (Figure 2).

### Unique transcript profiles of PBMCs in the individuals with tuberculosis infection and active disease

We next focused our analysis on identifying unique gene expression signatures of tuberculosis infection (TB&LTBI) and active disease (TB). We compared gene expressions of PBMCs following PPD stimulation between (1) TB&LTBI groups and HC group (TB&LTBI/HC) according to whether individuals was infected by MTB or not and (2) TB group and LTBI&HC groups (TB/LTBI&HC) according to whether active disease was clinically developed or not.

The results showed that 75 differentially expressed genes were identified in TB&LTBI/HC, and 249 genes were found in TB/LTBI&HC (Table 1). Most of the genes with ratio >4 belonged to signaling molecules and immune response molecules (Table 3). As shown in Figure 1B, 20 differentially expressed genes were shared between these two comparisons above, while 73% (55/75) and 92% (229/249) genes were specifically present in each comparison, respectively. Thus, 55 and 229 genes correspondingly stood for the specific transcript profiles associated with tuberculosis infection (TB&LTBI) and active disease (TB). Functional annotation of the 55 genes in tuberculosis infection profile revealed that gene cluster of regulation of T cell homeostatic proliferation was significantly enriched with increased expression levels in TB&LTBI compared with HC. Meanwhile, gene categories associated with taxis and response to external stimulus were enriched in active disease profile (corrected *P* value <0.05; Table S1).

**Table 3:** Significantly regulated genes in PPD-stimulated PBMCs unique for tuberculosis infection and active disease with ratio >4.

GeneSymbol	Means		Ratio	Regulation	t-test
**TB&LTBI/HC**	**TB&LTBI**	**HC**			***P*** ** value**
IL2	96.54	5.65	17.09	up	0.0005
CXCL9	10.39	1.32	7.86	up	0.0061
IFNG	13.17	1.76	7.49	up	0.0006
CXCL10	4.67	0.77	6.05	up	0.0096
CXCL11	3.81	0.65	5.85	up	0.0400
CCL8	3.89	0.70	5.57	up	0.0199
BATF2	4.05	0.84	4.80	up	0.0029
**TB/LTBI&HC**	**TB**	**LTBI&HC**			***P*** ** value**
BX090755	0.10	0.98	10.13	down	0.0050
IFNG	24.46	3.53	6.93	up	0.0014
UBD	8.01	1.38	5.81	up	0.0126
CXCL11	6.64	1.19	5.58	up	0.0474
CXCL9	16.42	2.95	5.57	up	0.0333
CXCL10	7.48	1.50	4.99	up	0.0267
RSPO3	1.76	0.36	4.85	up	0.0208
CCL8	6.22	1.30	4.77	up	0.0397
DB513559	0.09	0.37	4.38	down	0.0110
HMGA2	4.97	1.22	4.06	up	0.0001

### Validation of differentially expressed genes in independent sample set by qPCR

In order to validate the microarray results, we selected 30 differentially expressed genes from three pair-wise comparisons and 22 genes specific for tuberculosis infection and active disease to carry out the qPCR study using the same samples in the microarray experiment. A high concordance of qPCR results with microarray data was found (94%), which validates the reliability of the microarray study.

**Figure 3:**
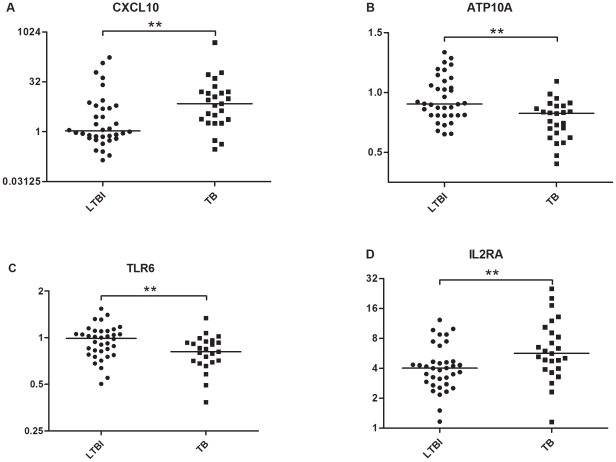
Scatter plots of four discriminatively expressed genes between TB and LTBI by qPCR with *P* value <0.01. Horizontal bar, median fold change of each group following PPD stimulation. ** significant difference: 0.001< *P* value <0.01.

We subsequently recruited additional 83 individuals: 25 with TB, 36 with LTBI and 22 with HC to further validate the microarray results using the same genes of interest above. As high as 81% of selected genes were confirmed by qPCR, which displayed the same regulation patterns as microarray study (Table S2). Genes with significantly differential expression among corresponding comparisons were shown in bold in Table S2 (Mann-Whitney U test, *P* value <0.05). Among these genes, IFN-γ, CXCL10, IL2 and CXCL11 exhibited statistically significant difference with *P* value <0.001 and ratio >2 in the comparisons of TB vs. HC, TB&LTBI vs. HC, and TB vs. LTBI&HC. Five genes (CXCL10, ATP10A, TLR6, IL2RA, and FLNB) were differentially expressed between TB and LTBI group and the fold changes of them in PPD-stimulated PBMCs from TB patients were greater than those from the LTBI individuals (Table S2). Scatter plots of four genes with *P* value <0.01 (CXCL10, ATP10A, TLR6, IL2RA) were shown in Figure 3.

### Identification of biomarkers to distinguish TB from LTBI

Both T-SPOT.TB and QuantiFERON-TB Gold In-tube assays that detect antigen specific IFN-γ release cannot discriminate TB patients from individuals with latent infection [15]. Our study also indicated that the expression levels of IFN-γ was not significantly different between TB and LTBI by qPCR (*P* value  = 0.07; Table S2). Thus, we attempted to discover whether multiple genes possessed better predictive power than any single gene in discriminating TB from LTBI.

**Figure 4:**
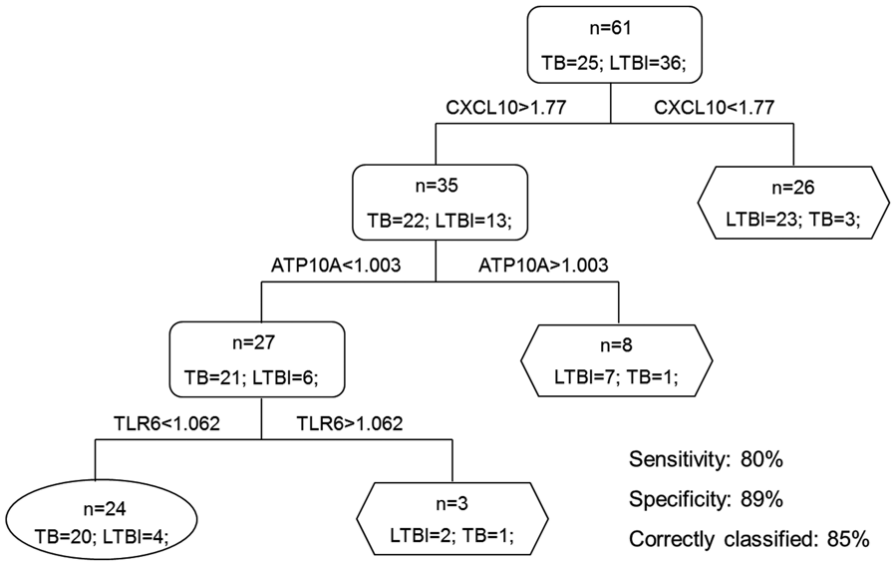
The combination of CXCL10, ATP10A and TLR6 provides the best discrimination between TB patients and LTBI individuals. The sensitivity and specificity of this three-gene panel was 80% and 89% respectively. 85% individuals were correctly classified. TB group, n = 25; LTBI group, n = 36. Rectangle: internal nodes; Oval and hexagon: terminal nodes showing the number finally determined as TB and LTBI, respectively.

Receiver operating characteristic (ROC) methodology was applied to evaluate the discriminatory ability of five differentially expressed genes (CXCL10, ATP10A, TLR6, IL2RA, and FLNB) between TB and LTBI in microarray validation study. The values of area under curve (AUC) of these genes are shown in Table 4. Thereafter, we subjected these five genes to decision tree analysis to find ideal gene combination and to optimize the discrimination between TB and LTBI using R program with 15-fold cross-validation. This analysis demonstrated that a combination of CXCL10, ATP10A and TLR6 provided the best predictive capacity, with as many as 85% individuals being correctly classified (Figure 4). The sensitivity of this three-gene combination was 80%, as 20 of 25 TB patients were correctly identified; and the specificity was 89%, as only 4 of 36 LTBI individuals were incorrectly identified as active TB.

**Table 4:** ROC analysis of selected genes for discrimination between TB group and LTBI group.

Gene	AUC	*P* value	%Sensitivity	%Specificity	Sum
Microarray validation set (n = 61)					
ATP10A	0.74	0.0019	72	64	1.36
CXCL10	0.72	0.0045	88	64	1.52
TLR6	0.70	0.0099	88	50	1.38
IL2RA	0.70	0.0073	72	78	1.50
FLNB	0.71	0.0442	64	72	1.36
Biomarker validation set (n = 36)					
ATP10A	0.72	0.0235	59	79	1.38
CXCL10	0.85	0.0004	65	95	1.59
TLR6	0.79	0.0034	76	74	1.50

ROC, receiver operating characteristic; AUC, area under curve; Sum, the sum of sensitivity and specificity.

We subsequently validated the diagnostic ability of this combination to identify TB patients from individuals with MTB infection in 36 subjects (TB = 17, LTBI = 19), whose clinical status was unknown to the investigators. The expression of three biomarkers of interest was examined by the same procedure as above. It turned out that there was significant difference of these three genes between TB and LTBI (Table S2). The ROC results were shown in Table 4. According to the optimal cutoff yielded by the decision tree analysis, the combination of CXCL10, ATP10A and TLR6 could correctly classify 81% individuals, with a sensitivity of 71%, specificity of 89%, positive predictive value of 86% and negative predictive value of 77% (Figure S1).

## Discussion

The human immune response to MTB infection is highly complex and multifaceted. Identifying the immunologic characteristics in different infectious status will facilitate development of interventions to control initial infection and prevent reactivation of latent infection. In this study, we described the global gene expression differences in TB, LTBI and HC, and identified distinct transcriptional signatures of tuberculosis infection and active disease. Most importantly, we found that the combination of CXCL10, ATP10A and TLR6 could be used as novel biomarkers to differentiate TB from LTBI.

Unlike most previous microarray studies on TB using non-stimulated whole blood [22], [23], [25], the present work examined transcriptional profiles using PPD-stimulated PBMCs to concentrate on detecting changes in immune response specific to MTB infection and therefore to identify TB-specific gene expression changes. To our knowledge, this is the first study using MTB-specific antigens as stimulus to activate PBMCs which are known to contain both monocytes and lymphocytes needed for recalling immune response and elucidate the complex immune responses present in different status of TB infection. PPD as a mixture of MTB antigens widely used in skin test, could stimulate more comprehensive cytokines spectrum than ESAT-6/CFP-10 used in IFN-γ release assays [20]. Therefore, the stimulation of PPD could provoke more diverse TB-specific immune responses than ESAT-6/CFP-10, allowing potentially more comprehensive immune factors to be included that may facilitate better distinction between TB and LTBI.

The individuals recruited were from BCG-vaccinated populations. Therefore, the LTBI individuals were defined as positive IGRAs test, independent of TST results [26]. In order to concentrate on detecting the immunological difference in response to MTB infection, we recruited healthy donors from the family members who lived together with the TB patients [23]. We also chose a relatively short stimulation time (t = 4 hours) to minimize the influence of other factors on PBMCs activation, in order to enhance the detection of genes expressed in early immune response to MTB-specific antigens [27], [28].

In the pair-wise comparisons, TB patients displayed the most pronounced difference compared with individuals from LTBI and HC. Functional annotation revealed that genes involved in response to external stimulus were enriched in all three pair-wise comparisons, which indicated that variance in the early behavior of PBMCs in response to PPD might contribute to the later immune consequence. In addition, several studies have been performed on the individuals with MTB infection to identify gene expression profiles [22], [23], [25]. Genes in the categories of chemotaxis, cell communication, signal transduction, inflammatory response and protein binding were identified in comparisons of TB vs. LTBI and TB vs. HC [23], [25], which is consistent with our data (Table S1). Maertzdorf *et al* suggested the importance of apoptosis regulation and human defense responses in the susceptibility and resistance to TB, the latter of which also exhibited significance in our study (Table S1). However, FCGR1B, identified as the most differentially expressed gene between TB and LTBI in also Maertzdorf's study, was not proven in our study as *P* value was 0.79. In another study by O'Garra and colleagues, a neutrophil-driven IFN (both IFN-γ and type I IFN-αβ)-inducible gene profile was identified as TB-specific signature [22]. Similarly, our data showed that signaling pathway of IFN-γ displayed significant difference with a corrected *P* value  = 0.0032 in the comparison between TB and LTBI, implicating its crucial role in immune response against MTB infection.

In the multivariate analysis, despite IL2RA exhibiting significant difference in expression between TB and LTBI by qPCR (*P* value  = 0.0073), it was excluded from the final diagnostic combination of CXCL10, ATP10A and TLR6. Spearman correlation analysis revealed that the correlation of expression between IL2RA and CXCL10 was significant with R = 0.63 and *P* value <0.0001, indicating their close association and similarity in expression pattern for both LTBI and TB conditions, and therefore inclusion of IL2RA into the diagnostic combination is unlikely to improve the discriminative power of the test any further. Eventually, the measurement of PPD-stimulated changes in mRNA levels for CXCL10, ATP10A and TLR6 was validated as the best predictor distinguishing TB from LTBI, with 71% sensitivity, 89% specificity, and 81% of individuals correctly classified. Its discriminative capacity was comparable to single-positive TNF-α MTB-specific CD4^+^ T cells or the combination of RAB33A, CD64 and LTF [25], [29]. The present study indicates that this three-gene panel possesses the capacity to serve as novel diagnostic biomarkers separating TB from LTBI.

In microarray validation study, there were four genes exhibiting remarkable difference in TB group compared with LTBI group with *P* value <0.01: ATP10A, CXCL10, TLR6 and IL2RA. ATP10A belonged to the family of P-type cation transport ATPases with a well-defined phosphorylation domain [30] and subfamily of aminophospholipid-transporting ATPases [31]. ATP10A was suggested to be imprinted and associated with type 2 diabetes [32]. Though other ATPases such as ATP1B3, ATP8B2, and ATP6V0E1 were reported to be differentially expressed genes between TB patients and LTBI individuals in the O'Garra study [22], ATP10A was for the first time found as a differentially expressed gene which was more repressed in TB patients than in LTBI individuals upon PPD stimulation (Table S2). ATP10A was reported to play roles in transmembrane movement of small molecules, but whether this function is related with TB susceptibility and resistance remains tentative.

CXCL10, also known as IP-10 (IFN-γ-induced-protein 10), is a CXC chemokine produced by several cell types such as monocytes and T cells, and is located on chromosome 4 in a cluster with CXCL9 and CXCL11. In the current study, the expression levels of these three genes (CXCL9, CXCL10 and CXCL11) did show a notable consistency (Table 2, 3). The binding of CXCL10 to receptor CXCR3 can contribute to pleiotropic effects, including stimulation of monocytes, natural killer and T-cell migration and modulation of adhesion molecule expression [33], [34]. Expression of CXCL10 has been reported in many Th1-type inflammatory diseases, through recruiting monocytes and activating T-cells into sites with tissue inflammation [35]. In addition, elevated levels of CXCL10 have been reported in plasma and infection foci in individuals infected with MTB [36], [37]. As a potential marker for the diagnosis of MTB infection, CXCL10 had been used to improve the sensitivity of IGRAs [37], [38], while few reports its performance in distingushing between TB and LTBI. Here in our study, CXCL10 exhibited good diagnostic power in discriminating TB from LTBI (Table 4). Its expression was highly up-regulated by PPD in TB patients, but not in individuals with LTBI in our microarray and biomarker validation study (Table S2). This strong up-regulation of CXCL10 in PPD-stimulated PBMCs from TB patients may reflect its active role in the inflammatory process involved in TB pathogenesis.

TLR6 variant was documented to contribute to human susceptibility to TB [39] and was up-regulated in fresh unstimulated whole blood of patients with active pulmonary TB (n = 10) compared with healthy donors [40]. In contrast, our data showed that TLR6 expression in PBMCs was down-regulated by PPD in TB patients whereas almost no change in LTBI individuals (Table S2). Such variance may result from the use of the stimulus of the specific antigens and needs to be further validated within a larger number of samples. Heterodimers of TLR2 with TLR6 have been reported to be responsible for the recognition of soluble factors of MTB [41], and this ligation of TLRs may trigger either MyD88-dependent or MyD88-independent pathways involving in the initiation of T-cell mediated immunity [42]. Given the important role of TLRs in innate immunity and initiation of adaptive immune response, the diverse regulation pattern of TLR6 expression in TB and LTBI was likely to be a factor responsible for the different clinical outcome following MTB infection, which needs to be future investigated.

IL2RA, also known as CD25, is the alpha chain of the IL2 receptor which is present on activated T cells and B cells [43], [44]. Antigen binding to the T cell receptor (TCR) stimulates the expression of IL2 and IL2R, which can subsequently stimulate the growth, differentiation and survival of antigen-selected cytotoxic T cells [45], [46], [47]. This feature may favor the elimination of intracellular MTB. IL2RA is also surface marker of regulatory T cells (Tregs). It was reported that Tregs could inhibit Th1 responses and its frequency was high in active tuberculosis compared with uninfected donors and individuals with latent infection [48], [49]. In our study, the expression level of IL2RA induced by PPD was also higher in TB than that in LTBI group.

In summary, we analyzed comprehensive gene expression patterns associated with different MTB infectious status, and confirmed that the combinations of CXCL10, ATP10A, and TLR6 could be used as reliable predictor for active TB. These findings would not only have important implications for developing novel diagnostic biomarkers to differentiate active disease from LTBI, but also shed light on immune mechanisms underlying human staying latency or progression to active disease after infection with MTB and the immunological determinants promoting this transformation.

## Materials and Methods

### Human subjects

Patients with active TB were diagnosed based on the following criteria: clinical signs and symptoms including fever, cough and productive sputum; a suggestive chest X-ray; positive Ziehl-Neelsen stain for acid-fast bacilli or positive culture for MTB in sputum. In order to avoid the influence of anti-TB treatment, patients who received directly observed short-course therapy (DOTS) more than 4 weeks were excluded. Patients with allergic diseases, diabetes, cancer, immune-compromised conditions, and co-infections with HBV were also excluded. LTBI subjects were recruited from close contacts of active TB patients, with positive T-SPOT.TB test (Oxford Immunotech, UK), negative chest radiograph and no clinical symptoms or evidence of active TB [26], [50]. Healthy controls were also recruited from family members [23] of patients with negative T-SPOT.TB test, no clinical evidence of TB and normal chest radiography. All subjects were >14 years of age and HIV-negative.

In the microarray study, twelve subjects were enrolled with three groups: TB (n = 4), LTBI (n = 4) and HC (n = 4). The qPCR validation study for microarray experiments enrolled 83 subjects including 25 TB patients, 36 LTBI individuals and 22 healthy controls, which is designated as ‘microarray validation set’. In order to estimate the diagnostic power of the three biomarkers, another validation set, termed as ‘biomarker validation set’, was recruited with 17 TB patients and 19 LTBI subjects. TB patients were recruited from Pulmonary Hospital in Chongqing, China. The demographic characteristics of the study populations were described in Table 5. The study was approved with written consent by the Ethics Committee of Huashan Hospital, Fudan University, and written informed consent was obtained from all the participants.

**Table 5:** demographic characteristics of the study populations.

Study groups		TB	LTBI	HC
**Microarray set(n = 12)**				
Number of subjects		4	4	4
Age years(median)		30(22–68)	37(19–53)	30(14–48)
Gender (male/female)		2/2	2/2	1/3
BCG vaccination	Yes	3	4	4
	No	0	0	0
	Not known	1	0	0
TST	+	2	4	0
	−	1	0	4
	Not known	1	0	0
**Microarray validation set(n = 83)**				
Number of subjects		25	36	22
Age years(median)		41(16–86)	50(14–83)	46(14–77)
Gender (male/female)		16/9	13/23	7/15
BCG vaccination	Yes	16	29	17
	No	7	5	3
	Not known	2	2	2
TST	+	10	26	11
	−	6	2	2
	Not known	9	8	9
**Biomarker validation set(n = 36)**				
Number of subjects		17	19	
Age years(median)		47(21–84)	52(30–76)	
Gender (male/female)		13/4	8/11	
BCG vaccination	Yes	13	16	
	No	1	1	
	Not known	3	2	
TST	+	12	11	
	−	0	5	
	Not known	5	3	

### Isolation and stimulation of PBMCs

Peripheral blood (4ml) was withdrawn from median cubital vein of the antecubital fossa from each participant in heparinized vacutainer tubes (Becton Dickinson). PBMCs were separated by density gradient using Lympholyte Cell Separation Media (CEDARLAN, Canada) within 6 hours of blood withdrawal. The number of trypan blue-stained cells was counted using Countess® Automated Cell Counter (Invitrogen, USA). PBMCs from each subject were divided into two portions and cultured with AIM-V (Invitrogen Life Technologies, USA) containing 2 mM L-glutamine, 50 µg/ml streptomycin sulfate, 10 µg/ml gentamicin sulfate, and stimulated with or without 10 µg/ml *Mycobacterium tuberculosis* purified protein derivative (PPD, Mycos Research LLC, Loveland, CO, Colorado, USA) for 4 hours before RNA extraction.

### RNA extraction

Total RNA was extracted from PBMCs using TRIzol reagent (Invitrogen Life Technologies) according to the protocols recommended by the manufacturer. RNase-free DNase I (Invitrogen Life Technologies) was used to remove genomic DNA contamination. The integrity and quality of RNA was evaluated by Agilent 2100 Bioanalyzer (Agilent Technologies). RNA with a 2100 RIN (RNA integrity number) ≥7.0 and 28S/18S >0.7 was used for the microarray study.

### Microarray hybridization and data processing

Microarray hybridization, scanning, and standardization were performed in Shanghai Biochip Co., Ltd using Agilent's Whole Human Genome Oligo Microarray (Agilent Technologies) which contains about 41,000 genes and transcripts. A two-color (Cy3 and Cy5) hybridization format was used for the microarray. RNA extracted from PPD-stimulated PBMCs was labeled with Cy3 (Cy3-dCTP), whereas the one from non-stimulated PBMCs was labeled with Cy5 (Cy5-dCTP). cDNA synthesis was carried out using the Agilent Fluorescent Direct Label kit according to the manufacturer's instructions. The cDNA samples with and without PPD stimulation from each individual were hybridized to the same slide [51]. The labeling and hybridization were performed according to protocols from the manufacturer [52], [53]. The signals were scanned using Agilent G2565BA microarray scanner and normalized before analysis. All the microarray data was MIAME compliant and had been deposited in GEO database with accession number: GSE27984.

### Quantitative real-time PCR analysis

Purified RNA was reverse transcribed to cDNA using PrimeScript® RT reagent Kit (TaKaRa) according to the manufacturer's protocol. qPCR was performed using SYBR^TM^ Green PCR Master Mix (TaKaRa) following standard conditions. PCR amplification was conducted in ABI 7500 Real-time PCR System (Applied Biosystems, Inc). The relative amount of expressed RNA was calculated by comparison with the expression of the housekeeping gene GAPDH using the 2^−△△Ct^ method [54].

### Data analysis

In the microarray study, the fold change of gene expression level altered by PPD stimulation was expressed as log2 transformed Cy3/Cy5 intensities for each probe. Differentially expressed genes were identified based on the *P* values <0.05 by Student's *t*-test and the fold change >2.0. Unsupervised two-way hierarchical clustering was performed to analyze gene expression patterns of multiple genes simultaneously in 12 subjects [22]. Gene Ontology (GO) analysis was applied to functional analysis of differentially expressed genes using GeneSpring software. The database was downloaded from the website of Gene Ontology (http://www.geneontology.org). The significance of the association between the genes and GO terms was measured by *P* value calculated by hypergeometric test. Benjamin–Hochberg correction was used for multiple GO term testing and the correlation between them (corrected *P* value). The above statistical analyses were performed using GeneSpring GX version 11.0 (Agilent Technologies).

In the qPCR study, Mann-Whitney U test was used to compare gene expression levels among groups using SPSS 16.0 software (SPSS Company, Chicago, IL) and difference was considered significant when *P* value was <0.05. ROC analysis was performed to determine the discriminative ability of selected genes to distinguish TB from LTBI with the overall accuracy assessed by AUC values [55]. Combinations of markers were identified by decision tree analysis via R 2.12.1 (R Foundation for Statistical Computing). Using this algorithm, the best tree was chosen as previous study [21].

## Supporting Information

Figure S1 Combination of CXCL10, ATP10A and TLR6 could distinguish TB patients and LTBI individuals. The sensitivity and specificity of this three-gene panel was 71% and 89% respectively. 81% individuals were correctly classified. TB group, n = 17; LTBI group, n = 19. Rectangle: internal nodes; Oval and hexagon: terminal nodes showing the number finally determined as TB and LTBI, respectively.
(TIF)

Table S1 GO analysis of differentially expressed genes.
(XLS)

Table S2 Validation results of 30 differentially expressed genes in pair-wise comparisons and 22 genes specific for tuberculosis infection and active disease in 83 subjects by qPCR.
(XLS)
