# Supplementary figures and images for: Novel Biomarkers Distinguishing Active Tuberculosis from Latent Infection Identified by Gene Expression Profile of Peripheral Blood Mononuclear Cells
Source: PLoS One. 2011 Aug 31;6(8):e24290. doi: 10.1371/journal.pone.0024290 (PMC3164189; doi:10.1371/journal.pone.0024290)

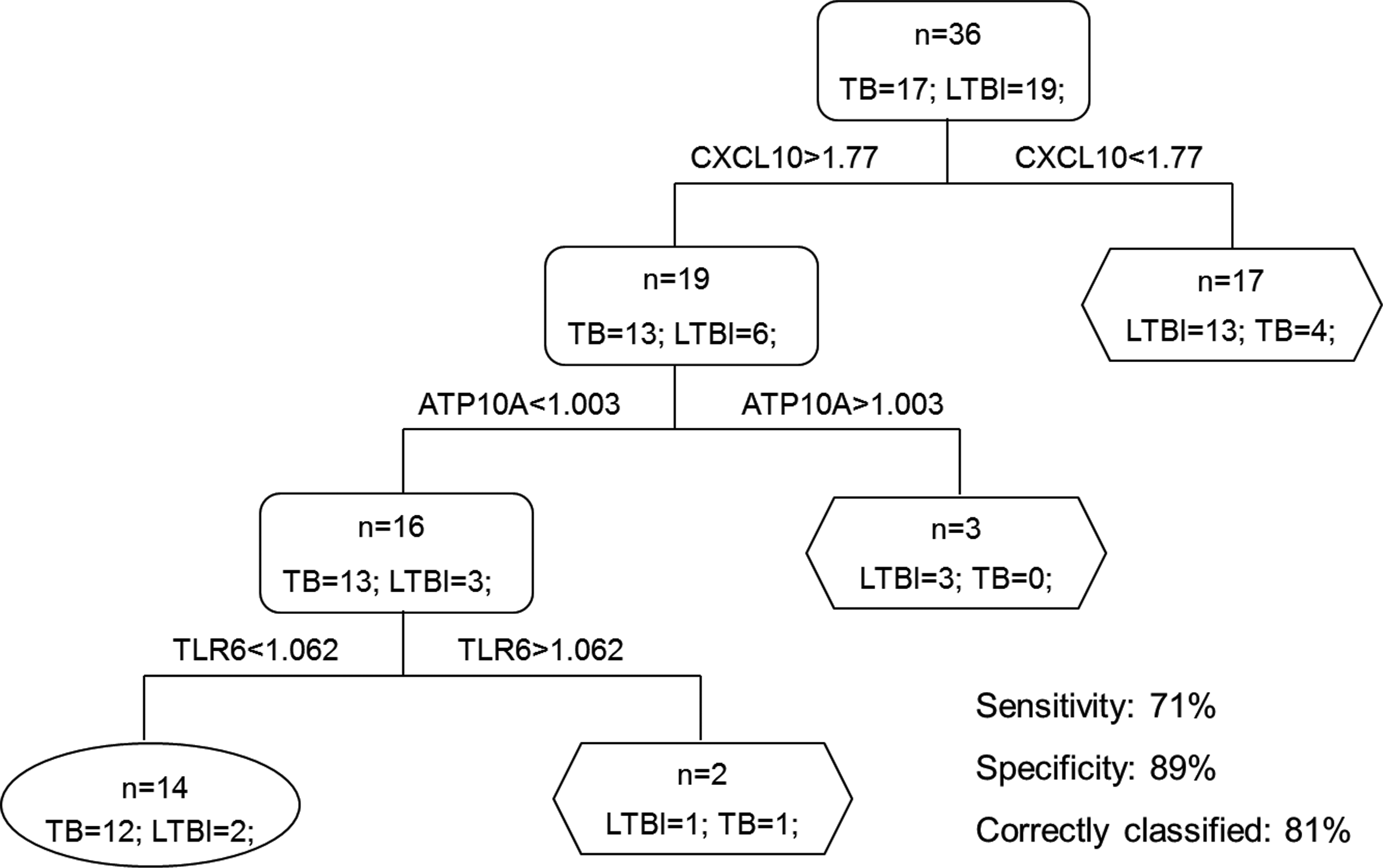

Supplement: Figure S1 — Combination of CXCL10, ATP10A and TLR6 could distinguish TB patients and LTBI individuals. The sensitivity and specificity of this three-gene panel was 71% and 89% respectively. 81% individuals were correctly classified. TB group, n = 17; LTBI group, n = 19. Rectangle: internal nodes; Oval and hexagon: terminal nodes showing the number finally determined as TB and LTBI, respectively. (TIF) [file pone.0024290.s001.tif]
